# Vitamin D in Photosynthetic Organisms and Fungi: Sterol Photochemistry, UV-B Availability, and Biofortification Potential

**DOI:** 10.3390/cimb48080760

**Published:** 2026-07-26

**Authors:** Ariam Abraham, Dorota Bartusik-Aebisher, Barbara Smolak, Klaudia Dynarowicz, Edward Kowalczyk, Wiesław Guz, David Aebisher, Gabriela Henrykowska

**Affiliations:** 1 English Division Science Club, Faculty of Medicine, University of Rzeszów, 35-310 Rzeszów, Poland; 2 Department of Biochemistry and General Chemistry, Faculty of Medicine, University of Rzeszów, 35-310 Rzeszów, Poland; 3 Department of Diagnostic Imaging and Nuclear Medicine, Faculty of Medicine, University of Rzeszów, 35-310 Rzeszów, Poland; bsmolak@ur.edu.pl (B.S.);; 4 Department of Pharmacology and Toxicology, Faculty of Medicine, Medical University of Lodz, T. Kosciuszki 4, 90-419 Lodz, Poland; 5 Department of Photomedicine and Physical Chemistry, Faculty of Medicine, University of Rzeszów, 35-310 Rzeszów, Poland; 6 Department of Epidemiology and Public Health, Faculty of Medicine, Medical University of Lodz, T. Kosciuszki 4, 90-419 Lodz, Poland

**Keywords:** vitamin D, secosteroids, UV-B radiation, sterol photochemistry, ergosterol, phytosterols, algae, fungi, biofortification, LC-MS/MS

## Abstract

Vitamin D comprises a group of fat-soluble secosteroids traditionally associated with animal physiology, calcium-phosphate homeostasis, and skeletal metabolism. However, vitamin D and related compounds have also been reported in taxonomically distinct non-animal systems, including fungi, microalgae, other algae, phytoplankton, and higher plants, although the strength of evidence differs substantially among these groups. This review synthesizes current knowledge on the occurrence, structural chemistry, UV-B-driven photochemical mechanisms, environmental determinants, analytical challenges, and biofortification potential of vitamin D formation in photosynthetic organisms and fungi. Vitamin D synthesis is initiated by UV-B radiation, primarily within the 290–315 nm range, which converts sterol precursors such as 7-dehydrocholesterol and ergosterol into previtamin D intermediates and is followed by thermal isomerization to the corresponding vitamin D forms. Continued irradiation may additionally generate lumisterol, tachysterol, and other photoproducts, thereby limiting net vitamin D accumulation. This non-enzymatic mechanism supports the interpretation that vitamin D formation can occur outside vertebrates when an appropriate 5,7-diene sterol precursor is accessible to a sufficient UV-B dose. In photosynthetic organisms and fungal matrices, net vitamin D accumulation is constrained by the spectral dose of UV-B, environmental exposure, tissue architecture, sterol localization, oxygen availability, antioxidant capacity, and ROS-mediated degradation. Studies of microalgae and phytoplankton, including reports concerning *Emiliania huxleyi*, suggest the occurrence or UV-B-dependent formation of both vitamin D_2_ and vitamin D_3_. However, these findings require evaluation according to the analytical method, use of authentic standards, experimental conditions, and confidence of compound identification. In fungi, the UV-B-induced conversion of abundant ergosterol to vitamin D_2_ is well established. Microalgae represent a developing source of vitamin D_2_ and vitamin D_3_, whereas evidence for nutritionally relevant vitamin D accumulation in higher plants remains limited and heterogeneous. Although higher plants contain diverse phytosterols, the formation of vitamin D_4_, vitamin D_5_, or related analogues requires appropriate photoreactive 5,7-diene precursors and should not be inferred directly from the presence of common phytosterols such as β-sitosterol. Analytical detection remains challenging because of low concentrations, complex lipophilic matrices, and structural similarity among secosteroids and photoproducts; therefore, reliable identification requires validated analytical procedures. LC-MS/MS provides high sensitivity and selectivity but should be supported by authentic standards, preferably isotope-labelled internal standards, retention-time agreement, quantitative and qualifying ions, matrix-recovery assessment, limits of detection and quantification, and evaluation of ion suppression. Structurally similar analogues and photoproducts may additionally require orthogonal confirmation. Nutritionally, post-harvest UV-B enrichment of edible mushrooms is currently the best-validated strategy for increasing non-animal vitamin D_2_ content. Microalgae constitute a developing platform for vitamin D_2_ and vitamin D_3_ production, whereas biofortification of higher plants remains experimental.

## 1. Introduction

Vitamin D is a fat-soluble secosteroid derived from sterol precursors and is recognized as essential for calcium and phosphate homeostasis in vertebrate organisms [1,2]. Structurally, vitamin D is defined by the photochemical rupture of the B-ring of the steroid nucleus between carbon atoms C9 and C10 generating an open-chain secosteroid with a conjugated triene system that confers characteristic photochemical reactivity and, after metabolic activation in vertebrates, biological activity [3]. The two principal biomedically relevant forms are vitamin D_3_ (cholecalciferol), synthesized in animal skin through UV-B-induced conversion of 7-dehydrocholesterol, and vitamin D_2_ (ergocalciferol), generated photochemically from ergosterol in fungi [1,4,5].

For many years, vitamin D was discussed mainly in the context of animal physiology, particularly skin synthesis, calcium-phosphate regulation, and skeletal metabolism [6]. This view has changed as analytical studies have reported vitamin D compounds and related sterol precursors in non-animal organisms. Reports from analytical chemistry and phytochemistry have identified vitamin D compounds or their sterol precursors in several photosynthetic organisms, including microalgae, macroalgae, and higher plants. However, the strength of this evidence differs among organism groups. UV-B-induced vitamin D_2_ formation from ergosterol is well established in fungi, evidence from microalgae is developing, and reports concerning higher plants remain comparatively limited and methodologically heterogeneous. Collectively, these observations suggest that vitamin D formation may occur outside animals when a suitable photoreactive sterol precursor is exposed to UV-B radiation. Proposed evolutionary interpretations of this chemistry remain hypothetical [4,5,6,7,8,9].

UV-B exposure is therefore a central determinant of vitamin D photochemistry, both in animal skin and in sterol-containing biological matrices. The biologically effective UV-B dose reaching terrestrial and aquatic surfaces is reduced and spectrally modified by atmospheric absorption and scattering, particularly by oxygen and ozone at wavelengths below approximately 290 nm [10,11,12,13,14].

Vitamin D-forming sterol photochemistry is driven primarily by UV-B rather than UV-A, whereas UV-C is largely removed before reaching the biosphere. The effective UV-B dose varies with latitude, season, solar zenith angle, altitude, cloud cover, atmospheric pollution, ozone concentration, and surface reflection. Vitamin D formation should therefore be considered a molecular photochemical reaction constrained by environmental UV-B availability [13,14,15,16].

In this review, photosynthetic organisms comprise microalgae, other algae, phytoplankton, photosynthetic microorganisms, and higher plants. Fungi are considered separately because they are neither plants nor photosynthetic organisms. Their inclusion is justified by the well-characterized UV-B-induced conversion of ergosterol to vitamin D_2_ and by the established use of this reaction in food enrichment. Photosynthesis and vitamin D photochemistry are mechanistically distinct: the former converts light energy into chemical energy, whereas the latter is a non-enzymatic UV-B-driven transformation of suitable sterol precursors [7,10].

Latitude, season, altitude, and surface albedo modify the natural UV-B dose available to exposed organisms. At higher latitudes, particularly during autumn and winter, an increased solar zenith angle reduces the amount of effective UV-B reaching terrestrial and aquatic environments. These factors may influence sterol photoconversion in naturally exposed algae, fungi, and plant tissues, although their effects depend on organismal exposure and matrix-specific conditions [11,12,17].

Principles established for cutaneous vitamin D_3_ synthesis are also relevant to vitamin D photochemistry in plants, algae, and fungi. In any biological matrix, UV-B photons must reach the appropriate sterol precursor before photoconversion can occur. In plants and algae, this photon access may be limited by tissue architecture, cuticular waxes, epidermal thickness, UV-absorbing pigments, flavonoids, hydroxycinnamic acid derivatives, and the spatial distribution of sterols within membranes. Thus, the presence of sterol precursors alone does not guarantee measurable vitamin D accumulation; the outcome depends on the interaction between UV-B exposure, precursor availability, tissue optical properties, temperature, oxygen availability, and competing oxidative degradation [7,18,19,20].

Despite growing interest in vitamin D from non-animal sources, significant knowledge gaps remain regarding the occurrence and possible physiological roles of vitamin D-related compounds in photosynthetic organisms, the identity of putative plant secosteroids, the contribution of fungal or endophytic contamination to some reported detections, and the nutritional relevance of the detected concentrations. This review aims to address these gaps systematically by integrating structural chemistry, photochemistry, environmental radiation science, analytical evidence assessment, and nutritional biofortification into a comprehensive framework [11,21].

This perspective is nutritionally important because vitamin D insufficiency remains a major global problem and dietary sources become increasingly relevant where natural UV-B-driven synthesis is seasonally or geographically limited. Among non-animal sources, post-harvest UV-B enrichment of edible mushrooms is the best-established strategy for increasing vitamin D_2_ content. A critical comparison of sterol precursor availability, UV-B exposure, analytical validation, product stability, and nutritional relevance is therefore required before reported vitamin D formation can be translated into reproducible food-production strategies [11,20,21,22,23].

## 2. Structural Chemistry of Vitamin D

Vitamin D compounds belong to the secosteroid family, a structurally distinct subclass of steroids in which one ring of the classical cyclopentanoperhydrophenanthrene steroid nucleus has undergone photochemical cleavage. Specifically, the B-ring of the steroid backbone is ruptured between C9 and C10 by UV-B irradiation, converting the rigid four-ring steroid architecture into a flexible, open-chain secosteroid. This structural transformation is the defining event in vitamin D formation and underlies the molecule’s characteristic physicochemical properties [1,3,24,25,26,27].

In biochemical terms, vitamin D belongs to fat-soluble steroid-derived compounds, which explains both its lipophilic behavior in biological matrices and its association with lipid-rich environments such as membranes, adipose tissue, and food matrices containing fats. Its fat solubility is also relevant analytically and nutritionally, because vitamin D and its precursors are extracted and quantified predominantly within lipid fractions rather than aqueous cellular components. After ingestion, vitamin D is also absorbed together with dietary lipids [28,29].

The biologically relevant vitamin D compounds are derived from structurally distinct sterol precursors and differ primarily in their C-17 side chain configuration. Vitamin D_3_, or cholecalciferol, C_27_H_44_O, with molecular weight 384.64 g/mol, is formed from 7-dehydrocholesterol, also known as provitamin D_3_, and is the predominant form synthesized in animal skin. Vitamin D_3_ or its precursor has also been reported in some algal systems, although the evidence remains developing and varies among species and studies. Its side chain is saturated, lacking additional double bonds or methyl substituents beyond C-20. Vitamin D_2_, or ergocalciferol, C_28_H_44_O, with molecular weight 396.65 g/mol, is derived from ergosterol, or provitamin D_2_, a principal membrane sterol in fungi. Structurally, vitamin D_2_ is characterized by an additional C22–C23 double bond and an extra methyl group at C24 relative to vitamin D_3_. These side-chain differences permit analytical distinction between the two compounds and contribute to their different metabolic behavior after ingestion [30,31,32,33,34].

The distinction between cholecalciferol and ergocalciferol is important because both compounds are biologically inactive precursors before metabolic activation, yet they originate from different sterol substrates and ecological sources. Cholecalciferol is associated mainly with UV-induced conversion of 7-dehydrocholesterol, whereas ergocalciferol is produced through UV irradiation of ergosterol, particularly in fungi and yeasts. This difference directly connects the structural chemistry of vitamin D with the biological source of the molecule: animal-derived, fungal-derived, algal-derived, or reported in higher-plant matrices [35,36,37,38,39].

Beyond the canonical D_2_ and D_3_ forms, additional vitamin D analogues have been described, although their occurrence and biological relevance are less firmly established. Vitamin D_4_, or 22-dihydroergocalciferol, arises from the photolysis of 22,23-dihydroergosterol, also termed provitamin D_4_*,* while vitamin D_5_, or sitocalciferol, is formed from 7-dehydrositosterol, a 5,7-diene derivative of β-sitosterol and the direct provitamin D_5_ precursor. The chemical feasibility of these transformations does not by itself establish that nutritionally relevant quantities of vitamin D_4_ or vitamin D_5_ accumulate naturally in higher-plant tissues. Evidence should therefore be evaluated separately for isolated precursor photolysis, detection in biological matrices, and confirmed occurrence in intact organisms. The principal vitamin D forms, their sterol precursors, source organisms, and distinguishing structural features are summarized in Table 1 [4,40,41].

This broader family of vitamin D analogues is especially relevant for the evaluation of sterol photochemistry in higher plants. Plant tissues contain diverse phytosterols, and not all of them correspond directly to animal cholesterol-derived pathways. Therefore, the structural diversity of phytosterols creates the possibility of multiple vitamin D-like secosteroids, including compounds whose nutritional or physiological significance remains uncertain. However, the presence of common phytosterols such as β-sitosterol does not demonstrate direct vitamin D formation. Photoconversion requires an appropriate 5,7-diene system, and reports of D_2_, D_4_, D_5_, or related compounds in higher plants require appropriate analytical confirmation [4,39,40,41].

The conjugated triene system formed after B-ring opening is a defining structural feature of vitamin D compounds. It participates in the thermal conversion of previtamin D to vitamin D and also contributes to further photochemical conversion into lumisterol, tachysterol, and other photoproducts. The same unsaturated system increases susceptibility to oxidative degradation by singlet oxygen and other reactive oxygen species [35,42,43].

Formation of vitamin D does not require a dedicated enzyme-catalysed pathway at the initial photochemical stage. The principal structural requirement is an accessible sterol precursor containing the appropriate 5,7-diene system, which can undergo UV-B-induced B-ring opening followed by thermal rearrangement. Consequently, the detection of abundant phytosterols without this photoreactive arrangement is not sufficient evidence for vitamin D formation [39,43].

Unlike rigid cholesterol and classical sterols, vitamin D molecules exhibit substantial rotational freedom around the broken B-ring. In vertebrates, this conformational flexibility contributes to interactions with vitamin D-binding protein and, after metabolic activation, with the vitamin D receptor and vitamin D-metabolizing enzymes. In plant systems, these receptor interactions are absent or uncharacterized, suggesting that vitamin D analogues generated in planta cannot currently be assumed to perform functions analogous to those established in vertebrates [30,44,45,46,47].

From the nutritional perspective, this structural difference between D_2_ and D_3_ may influence their binding, metabolism, and persistence after ingestion. However, detailed human pharmacokinetics fall outside the principal scope of this review and are considered only insofar as they affect the nutritional interpretation of fungal-, algal-, or plant-derived vitamin D [45,46,47].

Overall, the structural chemistry of vitamin D provides the molecular bridge between sterol biology, UV-B photochemistry, and nutritional relevance. In photosynthetic organisms and fungi the crucial issue is not merely the presence of vitamin D itself, but the availability of sterol precursors with the appropriate photoreactive architecture. Only sterols with an appropriate 5,7-diene system can function directly as vitamin D provitamins. The structural diversity of these substrates may contribute to variation in product identity, yield, stability, detectability, and possible biological significance; however, chemical plausibility must be distinguished from analytically confirmed occurrence in intact organisms [39,40,41,43].

## 3. Photochemical Mechanism of Vitamin D Synthesis

Vitamin D formation begins with a non-enzymatic photochemical reaction driven primarily by UV-B radiation within the approximately 290–315 nm range. The reaction can occur when a sterol containing an appropriate 5,7-diene system is accessible to a sufficient UV-B dose. This chemical mechanism is not restricted to vertebrate tissues, although its occurrence and efficiency must be evaluated separately in each biological matrix [43,48,49,50].

This UV-B dependence reflects the fundamental requirement for radiation of sufficient energy, especially within the range capable of interacting with sterol precursors such as 7-dehydrocholesterol and ergosterol. Accordingly, the extent of photoconversion depends on wavelength, UV-B dose, exposure time, precursor abundance, and the accessibility of the sterol within the biological matrix [49,51,52,53].

The two principal pathways of vitamin D formation the ergosterol-to-vitamin D_2_ pathway and the 7-dehydrocholesterol-to-vitamin D_3_ pathway follow the same fundamental photochemical logic. In fungi, ergosterol functions as provitamin D_2_ and is converted by UV-B irradiation into previtamin D_2_, which then isomerizes thermally to vitamin D_2_. This pathway is well established in fungi and yeasts; evidence in other matrices remains developing or limited, depending on the organism and analytical method. In animal skin and certain algal systems, 7-dehydrocholesterol functions as provitamin D_3_ and undergoes an analogous conversion to previtamin D_3_ and then cholecalciferol [54,55].

At the molecular level, UV-B absorption by the 5,7-diene system initiates cleavage of the C9–C10 bond in the sterol B-ring. This produces an open-chain previtamin D intermediate. Detailed excited-state and orbital-symmetry descriptions are not required to interpret the biological or technological significance of this reaction, but they confirm that the initiating step is photochemical rather than enzyme-catalysed [56,57,58,59].

The same structural requirement may apply in fungal, algal, and higher-plant matrices, but chemical plausibility should not be equated with confirmed formation in intact organisms. Reliable evidence additionally requires demonstration of the appropriate precursor and analytical confirmation of the resulting product [60,61,62,63].

The key structural event is UV-B-induced opening of the sterol B-ring between C9 and C10, producing previtamin D. This step may be described chemically as a conrotatory electrocyclic ring opening, but the essential biological point is that a suitable 5,7-diene precursor is converted into an open-chain secosteroid intermediate [60,64,65,66,67,68].

Previtamin D, the immediate photoproduct of ring opening, is a thermodynamically unstable intermediate that undergoes spontaneous thermal conversion to vitamin D. This step is temperature-dependent, and does not require enzymatic catalysis. In membrane environments, the lipid bilayer composition may influence the rate of this conversion [64,69,70,71].

Because this conversion is temperature-dependent, the final vitamin D yield may vary with tissue temperature, membrane environment, and post-irradiation conditions. These factors are relevant when comparing natural exposure with controlled UV-B treatment of fungal, algal, or plant material [69,70,71,72].

Under continued UV-B irradiation, previtamin D and vitamin D do not accumulate indefinitely but rather reach a photochemical equilibrium with photoproducts including lumisterol and tachysterol. Further irradiation may generate additional compounds, including toxisterols and other degradation products. This competing photochemistry limits vitamin D yield under prolonged or excessive exposure [73,74,75,76,77,78].

In naturally exposed or technologically irradiated matrices, the proportion of vitamin D relative to its photoproducts depends on the applied spectral dose and duration of exposure. Consequently, increasing UV-B treatment does not necessarily produce a proportional increase in vitamin D concentration [73,74,75,76,78].

In photosynthetic organisms, the photochemical formation of vitamin D occurs simultaneously with UV-B-induced generation of reactive oxygen species (ROS), including singlet oxygen (^1^O_2_), superoxide anion (O_2_^−^·), and hydroxyl radicals (·OH). The conjugated triene system of vitamin D is particularly vulnerable to oxidative degradation [79,80,81,82,83,84].

This oxidative dimension is highly relevant for plant and algal tissues because photosynthetic organisms naturally generate reactive oxygen species during light exposure. UV-B irradiation may therefore have a dual effect: it can initiate vitamin D formation from sterol precursors, but it can also intensify oxidative reactions that degrade either the precursors or the newly formed vitamin D compounds. Consequently, the final concentration of vitamin D in a plant, fungal, or algal matrix is not determined only by the amount of UV-B radiation, but also by the oxidative environment and the antioxidant capacity of the tissue.

The net accumulation of vitamin D in biological matrices represents a dynamic balance between UV-B-driven photochemical, formation from suitable sterol precursors and competing photodegradative processes, including singlet oxygen-mediated oxidation of the triene system and photochemical conversion to inactive lumisterol, tachysterol, and toxisterol photoproducts. The predominance of synthesis versus degradation depends critically on UV-B irradiance, tissue oxygen concentration, endogenous antioxidant capacity, sterol precursor availability, and membrane microenvironment. This balance may contribute to the low and variable concentrations reported in higher-plant and algal matrices; however, suitable photoreactive sterol precursors should not be assumed to be universally present [76,79,80,81,82,83,84,85].

In fungal, algal, and higher-plant matrices, this means that vitamin D production or enrichment cannot rely only on increasing UV-B dose. It must optimize irradiation wavelength, exposure duration, temperature, oxygen conditions, tissue structure, antioxidant status, and precursor availability in order to shift the system toward vitamin D accumulation rather than photochemical or oxidative loss [79,80,81,82,83,84,85].

## 4. Solar Radiation, UV-B Availability, and Environmental Determinants of Vitamin D Photochemistry

Solar radiation is the primary natural source of radiant energy reaching the Earth’s surface and therefore represents the environmental foundation for UV-B-driven vitamin D photochemistry. At the upper boundary of the Earth’s atmosphere, the solar irradiance incident on a surface perpendicular to the Sun’s rays is approximately 1360–1367 W·m^−2^, a value commonly referred to as the solar constant. This value varies slightly with the Earth–Sun distance, but it provides a useful reference point for understanding the amount of electromagnetic energy available before atmospheric filtering occurs [86,87].

The electromagnetic spectrum of solar radiation reaching the boundary of the atmosphere includes ultraviolet radiation, visible light, and infrared radiation. Ultraviolet radiation occupies the shorter-wavelength, higher-energy region of the solar spectrum; visible light spans approximately 380–780 nm; and infrared radiation extends beyond the visible range into longer wavelengths. In energetic terms, ultraviolet radiation is especially important for photobiology because its photons possess sufficient energy to drive electronic excitation and photochemical transformations in biological molecules, including sterol precursors of vitamin D [87,88].

As solar radiation passes through the atmosphere, it undergoes absorption, reflection, and scattering by atmospheric gases, water vapor, ozone, aerosols, dust particles, clouds, and surface-reflected components. Under conditions of high atmospheric transparency and near-vertical solar incidence, a large fraction of total solar radiation reaches the Earth’s surface, but only a small proportion of this energy lies within the ultraviolet range. Atmospheric absorption is particularly strong at shorter ultraviolet wavelengths, meaning that radiation below approximately 290 nm is largely filtered before it reaches terrestrial and aquatic biological systems [12,87].

This atmospheric filtering is critical for vitamin D formation because vitamin D photochemistry depends specifically on UV-B radiation. The photochemical conversion of sterol precursors such as 7-dehydrocholesterol and ergosterol occurs principally within the UV-B range of approximately 290–315 nm, the same spectral region required to initiate the electronic excitation that precedes electrocyclic B-ring opening. Thus, the availability of vitamin D-effective radiation at the Earth’s surface is not determined by total sunlight alone, but by the amount of biologically effective UV-B that survives atmospheric attenuation [89].

Ultraviolet radiation is conventionally divided into three bands: UV-A, UV-B, and UV-C. UV-A, approximately 315–380 or 315–400 nm depending on the classification system, is weakly absorbed by atmospheric gases and therefore represents the dominant ultraviolet component reaching the Earth’s surface. Although UV-A penetrates biological tissues more deeply and contributes to photoaging and oxidative stress, it is not the principal wavelength range responsible for vitamin D synthesis. UV-C, approximately 100–280 nm, has the highest photon energy and is strongly damaging to nucleic acids such as DNA and RNA, but it is almost completely absorbed by oxygen, ozone, and water vapor in the atmosphere. UV-B, approximately 280–315 nm, is only partially transmitted through the atmosphere and is therefore the limiting spectral component for natural vitamin D photochemistry [90].

In practical terms, only a fraction of incident UV-B reaches the Earth’s surface. Its intensity depends on season, time of day, latitude, solar zenith angle, altitude, atmospheric ozone concentration, cloud cover, aerosol load, air pollution, and local surface reflection. UV-B is absorbed primarily by stratospheric ozone, especially within the ozone layer located approximately 20–30 km above the Earth’s surface. Because of this strong ozone dependence, even modest variations in stratospheric ozone concentration may substantially modify the UV-B dose available for vitamin D-forming photochemical reactions [89,91].

The spectral composition of sunlight changes substantially between the top of the atmosphere and the Earth’s surface. Measurements and solar-spectrum models distinguish between extraterrestrial radiation, often described as AM0, and radiation reaching the ground after passage through the atmosphere, such as AM1 or AM1.5 spectra. During this passage, part of the solar energy is reflected back to space, while another part is absorbed by atmospheric oxygen, ozone, carbon dioxide, water vapor, and suspended particles. Additional scattering, including Rayleigh scattering and scattering on aerosols or dust, further modifies the intensity and spectral quality of the radiation that finally reaches biological surfaces [87,92].

This distinction is important because total solar energy may be high even when the biologically effective UV-B fraction is low. Visible and infrared radiation can dominate the total irradiance, while the biologically critical UV-B component may remain limited by ozone absorption, solar angle, atmospheric pollution, or cloud-related attenuation. Therefore, total sunlight, air temperature, or visible brightness cannot be used as reliable proxies for vitamin D-effective UV-B availability [89,91].

A “vitamin D-effective” UV-B window is central to plant, fungal, and algal vitamin D formation. In all of these systems, photons must be energetic enough to excite the sterol precursor but not so excessive in dose that photodegradation, oxidation, and conversion to inactive photoproducts dominate. Consequently, the same UV-B radiation that initiates vitamin D synthesis can also promote formation of lumisterol, tachysterol, toxisterols, and oxidized degradation products if exposure is prolonged or poorly controlled [74,76].

The photochemical efficiency of vitamin D synthesis in plants and algae depends critically on the UV-B irradiance spectrum, fluence rate, duration of exposure, and the spectral quality of incident light. Geographic latitude, season, time of day, cloud cover, and atmospheric ozone column all modulate UV-B fluence reaching plant surfaces. Additionally, leaf surface characteristics including cuticular wax composition, epidermis thickness, and presence of UV-absorbing flavonoids and hydroxycinnamic acid derivatives function as optical filters that attenuate UV-B penetration into mesophyll cells where sterol-rich membranes reside [93,94,95].

### 4.1. Latitude, Season, and Solar Zenith Angle

Latitude and solar angle are among the most important determinants of UV-B availability. At lower latitudes, the Sun reaches higher elevations above the horizon, and solar radiation travels through a shorter atmospheric path before reaching the surface. At higher latitudes, especially during autumn and winter, the solar zenith angle increases, the atmospheric path lengthens, and UV-B attenuation becomes much stronger. This creates seasonal and geographical variation in the capacity for UV-B-driven sterol photochemistry [89,92].

Seasonality further modifies UV-B availability. During summer, higher solar elevation and longer day length increase the daily window during which vitamin D-effective UV-B wavelengths reach the surface. During winter, lower solar elevation, shorter day length, and stronger atmospheric attenuation may reduce UV-B to levels insufficient for efficient photochemical conversion of sterol precursors. This seasonal limitation is often described as a “vitamin D winter,” especially in regions located at higher latitudes [89,96].

Comparable seasonal effects may influence naturally exposed fungal, algal, and higher-plant matrices. However, the presence of ergosterol, 7-dehydrocholesterol, or another suitable 5,7-diene precursor must be demonstrated rather than inferred from total sterol content. Higher summer UV-B may increase photoconversion, but it may simultaneously intensify photoproduct formation and oxidative degradation [74,95].

The solar zenith angle determines the optical path that ultraviolet photons must travel through the atmosphere. When the Sun is high in the sky, especially around solar noon, the path through the atmosphere is shorter and UV-B intensity is higher. When the Sun is low above the horizon, the same radiation passes through a longer atmospheric column, increasing absorption and scattering and reducing the biologically effective UV-B dose. This explains why UV-B intensity is highest in equatorial regions, during summer, under clear skies, and around midday [92,97].

Exposure timing is therefore critical for vitamin D photochemistry. Morning and late-afternoon sunlight may contain substantial visible and infrared radiation, but their UV-B component is usually lower because of the larger solar zenith angle. As a result, the same duration of exposure may produce different photochemical effects depending on exposure timing. In plants, fungi, and algae, this can influence whether sterol precursors are efficiently converted into vitamin D-like secosteroids or whether the UV-B dose remains below the effective threshold [92,97].

### 4.2. Altitude, Ozone, and Atmospheric Composition

Altitude further modifies UV-B availability. As elevation above sea level increases, the atmospheric column becomes thinner and less radiation is absorbed or scattered before reaching the surface. UV intensity increases on average by approximately 6–12% for every 1000 m increase in altitude, depending on the dataset and atmospheric conditions. This effect may be relevant not only for human skin exposure but also for high-altitude ecosystems, mountain plants, exposed lichens, fungi, and algal communities that experience stronger ultraviolet irradiation than comparable organisms at sea level [92].

Altitude should not be interpreted simply as increasing synthesis. Stronger UV-B at elevation could increase the probability of sterol photoconversion, but it may also enhance ROS formation, photoproduct accumulation, and oxidative degradation. Therefore, altitude may shift the entire synthesis–degradation balance rather than simply increase vitamin D accumulation [83,84].

Atmospheric ozone is one of the strongest regulators of UV-B penetration. Radiation below approximately 290 nm is largely absorbed before reaching the Earth’s surface, and UV-B radiation is absorbed mainly by stratospheric ozone. Because vitamin D photochemistry depends on wavelengths close to this atmospheric transmission boundary, even modest changes in ozone concentration can significantly alter the amount of vitamin D-effective UV-B reaching biological surfaces [91].

The protective ozone layer has undergone historical and regional changes, including episodes of reduced ozone thickness and so-called “mini-holes” over some areas, including parts of Central Europe. These events illustrate that regional UV-B availability cannot be inferred from latitude alone and may vary over relatively short time intervals [91].

Cloud cover, fog, aerosols, and air pollution further reshape both the intensity and spectral composition of UV radiation. Dense cloud layers can substantially reduce direct UV radiation, whereas thin or broken clouds may attenuate UV only partially. When diffuse radiation is considered, the total biologically relevant UV dose may remain significant even under conditions that do not appear strongly sunny [89,98].

Aerosols, dust particles, water droplets, and atmospheric pollutants scatter and absorb ultraviolet radiation. Urban or industrial pollution may therefore reduce the amount of UV-B reaching exposed organisms, while cleaner high-altitude or rural environments may allow stronger UV-B transmission. This means that two locations at the same latitude can differ substantially in vitamin D-effective UV-B availability because of local atmospheric composition [91,98].

### 4.3. Surface Reflection and Local Exposure

Surface reflection, or albedo, adds another environmental layer to UV-B exposure. Most natural surfaces, including grass and soil, reflect less than approximately 10% of incident UV radiation. Dry sand may reflect around 15%, water around 20%, and snow up to approximately 85% under certain conditions. This reflected component can increase total UV exposure in open environments, coastal ecosystems, snow-covered landscapes, and high-altitude regions. For low-growing plants, exposed fungi, lichens, intertidal algae, and other organisms located close to reflective surfaces, albedo may contribute meaningfully to the total UV-B dose reaching sterol-containing tissues. However, reflectance values vary according to surface composition, moisture, angle of incidence, wavelength, and local geometry; they should therefore be treated as approximate environmental modifiers rather than fixed predictors of vitamin D formation [92].

### 4.4. Temperature, Oxidative Conditions, and Matrix Accessibility

Temperature plays a dual role because it influences both the thermal conversion of previtamin D to vitamin D and the kinetics of competing degradation reactions. At low temperatures, thermal isomerisation may proceed more slowly, whereas higher temperatures may accelerate both conversion and oxidative loss. Tissue water content, oxygen availability, and the lipophilic microenvironment surrounding sterol molecules further modify the final photochemical outcome. The direction and magnitude of the temperature effect depend on the matrix and post-irradiation conditions; temperature should therefore be considered together with exposure time, membrane organisation, oxygen availability, and product stability [69,74].

Photosynthetically active organisms generate ROS as by-products of photosynthetic electron transfer, photorespiration, and other light-dependent reactions. Under UV-B exposure, additional ROS, particularly singlet oxygen, may be generated through photosensitisation involving chlorophylls, carotenoids, flavonoids, and other chromophores. These species can oxidise sterol substrates or newly formed vitamin D compounds and thereby compete with net vitamin D accumulation. In higher-plant and algal matrices, the measured vitamin D concentration may therefore reflect the balance between precursor photoconversion, subsequent thermal isomerisation, competing photoproduct formation, and oxidative degradation. The quantitative importance of these processes remains insufficiently characterised across biological matrices [83,84,85].

These variables matter because vitamin D formation in algae, fungi, and higher plants depends on UV-B photons reaching sterol-rich compartments. In aquatic systems, UV-B must penetrate the water column and reach phytoplankton or macroalgal tissues. In terrestrial plants, UV-B must pass through surface structures such as cuticular waxes, epidermal tissues, pigments, flavonoids, and phenolic compounds before reaching sterol-containing membranes. In fungi, UV-B exposure depends strongly on surface orientation, tissue geometry, pigmentation, moisture, and post-harvest irradiation conditions [93,94,95].

UV-B exposure should therefore be treated as a layered environmental variable rather than a simple measure of sunlight. At the planetary level, it is regulated by solar geometry, atmospheric filtering, and ozone. At the landscape level, it is modified by altitude, clouds, aerosols, and surface reflection. At the organismal level, it is controlled by tissue exposure, optical barriers, pigmentation, hydration, and the location of sterol precursors within biological structures. Each of these levels can either facilitate or limit vitamin D photochemistry [89,92,98].

Collectively, environmental variables determine the incident UV-B dose, whereas tissue and matrix characteristics determine how much of that dose reaches the precursor and whether the resulting product is retained or degraded. The measured outcome is therefore governed by both external exposure and internal matrix conditions [74,79,80,81,82,83,84,85].

From the perspective of biofortification, natural sunlight is often too variable to provide reproducible vitamin D yields in plant, algal, or fungal matrices. Controlled UV-B irradiation offers a way to standardize wavelength, dose, exposure time, temperature, and post-exposure handling, thereby shifting the balance toward predictable conversion of sterol precursors into vitamin D compounds. However, even in controlled systems, UV-B exposure must be optimized to avoid excessive photodegradation, oxidative loss, tissue damage, or accumulation of inactive photoproducts [74,95].

For edible fungi, this procedure is more precisely described as post-harvest UV-B enrichment when irradiation is applied after harvest. For microalgae and higher plants, the term biofortification should be used according to whether vitamin D content is increased during biological production or through a subsequent technological treatment.

The presence of sunlight alone does not ensure vitamin D formation. Effective photoconversion requires an appropriate UV-B spectral dose, accessible sterol precursors, and matrix conditions that limit photochemical and oxidative degradation [89,92,95].

## 5. Evidence for Vitamin D Formation Across Algae, Fungi, and Higher Plants

Vitamin D-related compounds have been reported in several taxonomically distinct non-animal systems. This section evaluates three groups separately: algae and phytoplankton, fungi, and higher plants. Particular emphasis is placed on sterol precursor availability, UV-B-dependent photoconversion, analytical confidence, and the standardized evidence category supporting vitamin D occurrence in each group [99,100,101,102,103].

### 5.1. Algae and Phytoplankton

Microalgae represent a potentially important photosynthetic source of vitamin D-related compounds. Holick [1] first proposed that phytoplankton may have been among the earliest organisms to synthesize vitamin D precursors, predating the evolution of terrestrial organisms by hundreds of millions of years. This evolutionary interpretation remains hypothetical but provides a framework for considering sterol photochemistry in ancient marine photic zones [1,99,100].

The marine coccolithophore *Emiliania huxleyi* has emerged as a key model organism for studying vitamin D synthesis in photosynthetic microalgae. Experimental UV-B irradiation of *E. huxleyi* cultures has been associated with the detection or increased formation of both vitamin D_2_ and vitamin D_3_. These findings challenge the traditional association of vitamin D_3_ exclusively with animals, but their interpretation depends on the analytical method, use of authentic standards, experimental controls, and confidence of compound identification. Detailed biochemical studies of *E. huxleyi* and other phytoplankton species, including Nannochloropsis and Chaetoceros, have identified ergosterol and related provitamins capable of UV-B photolysis [101,102].

Nutritional observations also support the relevance of microalgae. Fish are among the richest dietary sources of vitamin D partly because their food chains involve the accumulation of microalgae and plankton, which may contain vitamin D and previtamin D_3_. This observation supports the hypothesis that marine microorganisms may contribute directly or indirectly to the transfer of vitamin D-related compounds through aquatic food webs, although the quantitative contribution of individual taxa remains uncertain [103,104].

Brown algae, Phaeophyta, and green algae, Chlorophyta, have been reported to contain ergosterol and 7-dehydrocholesterol in variable quantities, providing potential substrates for vitamin D synthesis. Analytical studies using LC-MS/MS have detected vitamin D_3_ and 25(OH)D_3_ in several marine macroalgal species, with concentrations strongly correlated with UV exposure intensity and season. Reports concerning red algae are more limited, and the occurrence of vitamin D analogues in these taxa requires method-specific evaluation. Where 7-dehydrocholesterol or another suitable 5,7-diene precursor is present and accessible, UV-B-driven conversion to previtamin D_3_ and subsequently vitamin D_3_ is chemically plausible. However, the location of this process within specific algal membrane layers should not be assumed without direct experimental evidence [102,105].

Because algae occupy aquatic photic zones, their exposure to vitamin D-effective UV-B depends on water depth, transparency, dissolved organic matter, suspended particles, and seasonal solar angle. These environmental constraints are consistent with the broader geographical and atmospheric determinants described earlier, where UV-B availability is controlled by latitude, season, cloud cover, ozone, and scattering processes. In algal systems, however, these atmospheric variables are compounded by water-column attenuation, making actual sterol photoconversion dependent on both solar radiation reaching the surface and UV-B penetration into the aquatic habitat [100,105].

Overall, evidence from algae is developing. Detection of a precursor sterol, detection of a vitamin D compound in untreated biomass, and demonstration of increased product formation after controlled UV-B exposure represent different levels of evidence and should not be treated as equivalent.

### 5.2. Fungi as Non-Photosynthetic Vitamin D_2_-Producing Systems

Although fungi are neither plants nor photosynthetic organisms, they are important model systems for sterol-based vitamin D photochemistry. Ergosterol is a principal fungal membrane sterol and contains the 5,7-diene system required for UV-B-induced B-ring opening and formation of previtamin D_2_. Its abundance in many fungal tissues explains the efficient production of vitamin D_2_ following controlled UV-B exposure [106,107,108,109].

Fungal ergosterol conversion is mechanistically analogous to the UV-B-induced conversion of 7-dehydrocholesterol in animal tissues and selected algal systems. Both reactions involve photochemical B-ring opening followed by thermal isomerisation, but they yield different vitamin D forms because their sterol precursors differ structurally [108,109,110,111].

The commercial application of fungal ergosterol photochemistry is well established. Controlled UV-B irradiation of edible mushrooms, including *Agaricus bisporus*, *Pleurotus ostreatus*, and *Lentinula edodes*, can markedly increase vitamin D_2_ content. Reported concentrations range from very low values in mushrooms protected from sunlight to values exceeding 40,000 IU/100 g under particular experimental irradiation conditions. The magnitude of enrichment depends on species, tissue orientation, exposed surface area, UV wavelength and dose, moisture, temperature, and whether results are expressed on a fresh-weight, dry-weight, or serving-size basis. This post-harvest UV-B enrichment strategy is discussed in detail in Section 8 [108,112,113,114,115].

The ergosterol–vitamin D_2_ pathway is also governed by a photochemical equilibrium network involving previtamin D_2_, ergocalciferol, lumisterol, and tachysterol and further photoproducts. This network is analogous to the D_3_ pathway and explains why increasing UV-B dose does not produce an unlimited increase in vitamin D_2_ concentration [108,112].

Fungi therefore provide a useful model for understanding how precursor abundance, UV-B dose, exposure duration, and photochemical side-product formation interact. This also illustrates a central principle of this review: increasing UV-B exposure can increase vitamin D formation only up to a point, after which equilibrium reactions and degradation may limit further accumulation [112,114,115].

### 5.3. Higher Plants: Sterol Substrates and Photoproducts

Higher plants contain a diverse array of phytosterols that serve structural roles in cell membranes, modulate membrane fluidity, and participate in brassinosteroid biosynthesis. The principal phytosterols relevant to vitamin D photochemistry include β-sitosterol, the most abundant plant sterol, C_29_H_50_O; campesterol, C_28_H_48_O, a minor component with structural similarity to ergosterol; stigmasterol, C_29_H_48_O, containing a C22–C23 double bond analogous to ergosterol; and brassicasterol, C_28_H_46_O, found predominantly in Brassicaceae [113,116,117].

Common phytosterols such as β-sitosterol and campesterol do not themselves contain the 5,7-diene system required for direct vitamin D-forming photochemistry. Vitamin D_5_ is formed from the corresponding 5,7-diene precursor, 7-dehydrositosterol, rather than directly from β-sitosterol. Likewise, proposed formation of other phytosterol-derived secosteroids requires demonstration of a chemically appropriate provitamin precursor. The abundance of a common phytosterol alone is therefore not evidence of direct vitamin D formation.

Several higher-plant species from different families have been reported to contain vitamin D-related compounds, including *Solanum glaucophyllum*, *Cestrum diurnum*, *Trisetum flavescens*, and selected Solanaceae species. In *S. glaucophyllum* leaves, concentrations of approximately 3–7 μg/g dry weight have been reported for selected vitamin D_3_-related compounds, although the identified molecular forms and analytical methods vary among studies. Representative findings, analytical approaches, reporting bases, nutritional relevance, and standardized evidence categories are summarised in Table 2 [107,109,113,116].

Older reports based on animal bioassays or HPLC without tandem mass-spectrometric confirmation may establish biological activity or chromatographic similarity but do not necessarily provide compound-level identification equivalent to modern LC-MS/MS confirmation. Furthermore, reported D_2_ or ergosterol in higher-plant samples may reflect fungal, endophytic, or epiphytic contributions unless these potential confounders were specifically excluded.

The biological function of vitamin D or vitamin D analogues within plant tissues remains one of the most significant unresolved questions in plant biochemistry. Unlike in animals, plants possess no characterized vitamin D receptor, VDR, or vitamin D-activating hydroxylase enzymes, CYP27B1 or equivalent. Available evidence does not establish whether reported vitamin D-related compounds arise from regulated plant metabolism, incidental photochemistry, microbial associates, or a combination of these sources. Nevertheless, some researchers have proposed potential roles including modulation of sterol biosynthesis feedback, interaction with plant steroid hormone, brassinosteroid, signaling pathways, UV-stress response, and antimicrobial or antifungal functions. These hypotheses remain speculative and require direct functional evidence [113,116,117].

This distinction is important because the mere detection of vitamin D-like compounds in plants does not prove that they serve an animal-like hormonal function. Instead, their occurrence may reflect the same non-enzymatic sterol photochemistry described throughout this review. In higher plants, the final vitamin D profile will depend on phytosterol composition, UV-B exposure, tissue optical properties, antioxidant capacity, and the balance between photochemical synthesis and degradation [113,116].

Taken together, vitamin D accumulation in fungal, algal, and higher-plant systems is controlled by several interacting layers: external UV-B availability, tissue penetration, sterol precursor abundance, membrane microenvironment, temperature-dependent isomerisation, oxygen-mediated degradation, and antioxidant protection.

Using the standardized categories applied in Table 2, evidence is well established for UV-B-induced vitamin D_2_ formation in fungi, developing for vitamin D_2_ and vitamin D_3_ formation in algae, and limited for higher plants. The occurrence of a suitable precursor, adequate UV-B exposure, and a favourable matrix may permit photoconversion, but each reported product must be confirmed analytically rather than inferred from sterol composition alone [116,117].

**Figure 1:**
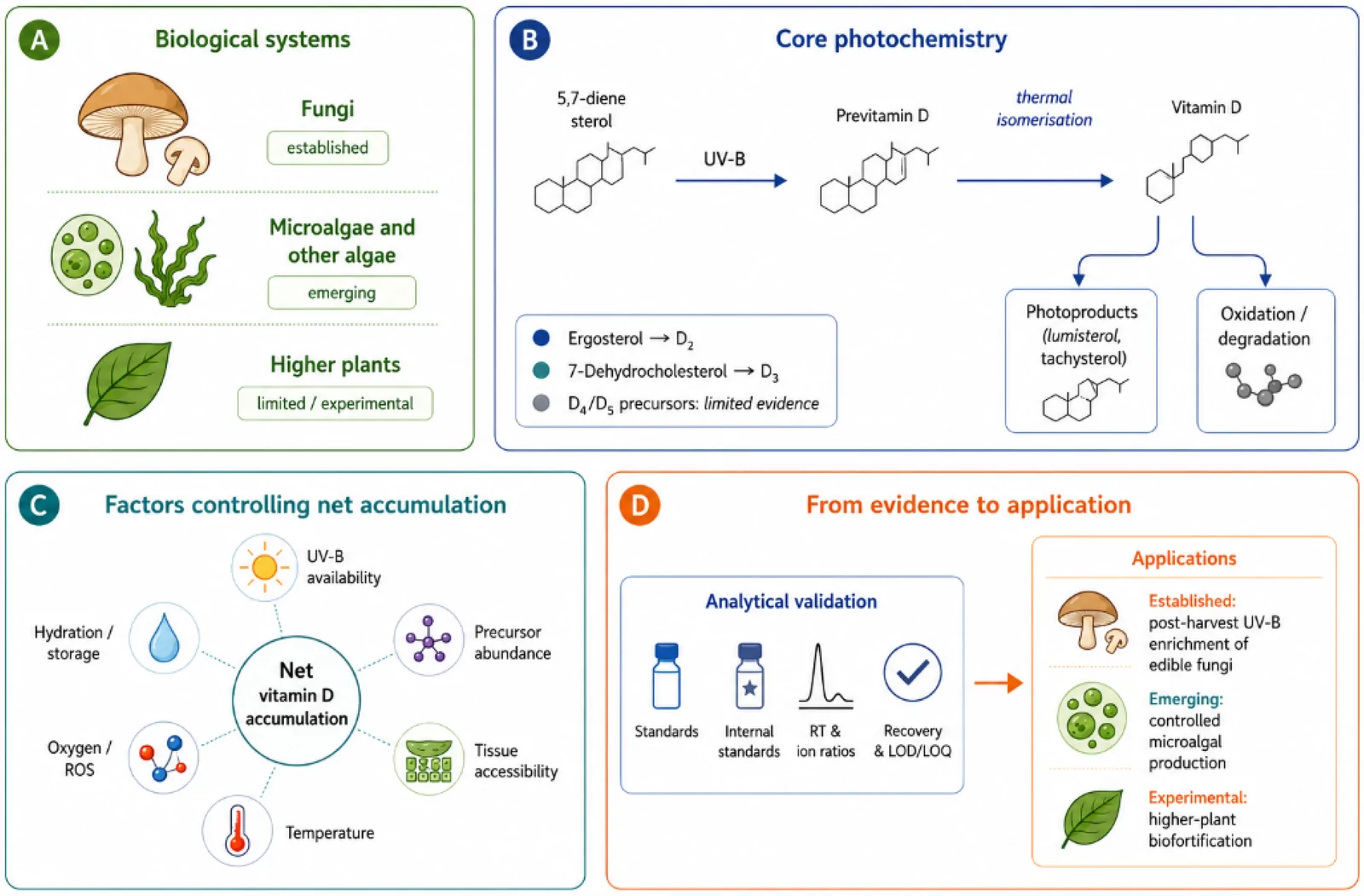
Integrated framework of UV-B-induced vitamin D formation, factors controlling net accumulation, and current applications in fungi, algae, and higher plants: (**A**) biological system; (**B**) Core photochemistry; (**C**) Factors controlling net accumulation and (**D**) From evidence to application.

An integrated overview of biological systems, core sterol photochemistry, factors controlling net vitamin D accumulation, and potential applications is presented in Figure 1.

## 6. Environmental and Tissue-Level Factors Modulating Accumulation

The photochemical efficiency of vitamin D formation in plants and algae depends critically on the UV-B irradiance spectrum (290–315 nm), fluence rate (W·m^−2^), duration of exposure, and the spectral quality of incident light. As discussed in Section 4, geographic and atmospheric variables determine the incident UV-B fluence reaching organismal surfaces. Additionally, leaf surface characteristics including cuticular wax composition, epidermal thickness, and the presence of UV-absorbing flavonoids and hydroxycinnamic acid derivatives function as optical filters that attenuate UV-B penetration into mesophyll cells where sterol-rich membranes reside [118,119,120].

Vitamin D photochemistry depends not only on external UV-B dose, but also on whether photons can reach the relevant sterol precursor inside a biological matrix. This principle is well illustrated by cutaneous vitamin D synthesis, where UV-B must penetrate to layers containing 7-dehydrocholesterol before previtamin D_3_ can be formed. In plant, algal, and fungal systems, the analogous constraint is tissue accessibility: UV-B radiation must reach ergosterol, 7-dehydrocholesterol, an appropriate phytosterol-derived 5,7-diene precursor, or other photoreactive sterols before photochemical ring opening can occur [119,121].

Temperature plays a dual role: it influences both the rate of thermal isomerization (previtamin D → vitamin D) and the kinetics of competing oxidative degradation reactions. At lower temperatures, thermal conversion may proceed more slowly, whereas higher temperatures may accelerate both isomerization and competing degradation reactions. Tissue water content, oxygen availability, and the lipophilic microenvironment surrounding sterol molecules further modulate photochemical outcomes [121,122].

Because vitamin D formation is not completed at the moment of photon absorption, irradiation and post-irradiation conditions must be considered together. The final concentration depends on the balance among photochemical precursor conversion, thermal isomerization, and product stability within the particular matrix [121,122].

Photosynthetically active organisms are inherently exposed to ROS generated as by-products of photosystem II electron transfer and photorespiration. In UV-B-irradiated tissues, additional ROS particularly singlet oxygen, are generated through photosensitization reactions involving chlorophyll, carotenoids, and flavonoids. These reactive species may compete with net vitamin D accumulation by oxidising sterol substrates or newly formed vitamin D compounds. This mechanism is chemically plausible and may contribute to the low and variable concentrations reported in higher-plant and algal matrices, although its quantitative importance has not been established systematically across these systems. The net vitamin D content in a photosynthetic matrix may therefore reflect the balance between photochemical formation and oxidative loss [123,124].

Matrix complexity influences both accumulation and analytical detection. Phytosterols, chlorophylls, carotenoids, flavonoids, tocopherols, cuticular lipids, and other lipophilic or antioxidant compounds may shield sterol precursors from UV-B photons, protect vitamin D from oxidative degradation, and interfere with extraction and quantification. Therefore, plant tissue should not be treated as a passive container for vitamin D formation, but as an active optical and chemical environment that shapes the final secosteroid profile. The analytical implications of matrix complexity and the requirements for reliable LC-MS/MS confirmation are discussed in detail in Section 7 [125,126,127].

Among experimental variables, UV-B dose and exposure duration have the most direct influence on accumulation. Insufficient irradiation may fail to convert a meaningful fraction of sterol precursors, whereas excessive irradiation may shift the photochemical network toward lumisterol, tachysterol, toxisterols, and oxidative degradation products. This is why controlled irradiation protocols must optimize dose rather than simply maximize exposure [128,129].

The relevant exposure variable is the absorbed spectral dose rather than irradiation time alone. Identical treatment durations may produce different outcomes depending on lamp spectrum, distance from the source, irradiance, sample orientation, exposed surface area, and optical properties of the material.

Sterol precursor availability is another major limiting factor. Biochemical descriptions identify 7-dehydrocholesterol as the immediate precursor for cutaneous vitamin D_3_ synthesis and ergosterol as the precursor for vitamin D_2_ formation in fungi and yeasts. By analogy, plant and algal vitamin D accumulation depends on the abundance, localization, and photoreactivity of their sterol pools. Differences in sterol abundance and photoreactivity may therefore contribute to variation in vitamin D accumulation among biological matrices [129,130].

Tissue architecture further modulates UV-B penetration. In higher plants, the cuticle, epidermis, wax layers, flavonoids, hydroxycinnamic acid derivatives, and other UV-absorbing compounds can reduce the amount of radiation reaching internal sterol-rich membranes. Thus, vitamin D photochemistry depends on biologically accessible UV-B: photons must not only reach the organismal surface, but also penetrate to sterol-containing compartments where photoconversion can occur [131].

Pigments and photoprotective compounds may have a dual effect. On one hand, they can protect tissues against excessive UV-B damage and ROS formation. On the other hand, they can decrease the effective photon flux reaching sterol precursors and thereby limit vitamin D synthesis. In photosynthetic organisms, chlorophylls, carotenoids, flavonoids, and related compounds therefore participate indirectly in regulating vitamin D accumulation by shaping both UV absorption and oxidative stress responses [123,131].

The net effect of these compounds may vary among species, tissues, developmental stages, and experimental conditions. A matrix with strong UV-screening capacity may exhibit limited precursor conversion despite high incident irradiation, whereas antioxidant-rich tissues may provide greater protection to the newly formed product.

Oxygen availability is also an important modifier. The photochemical formation of vitamin D requires UV-B exposure, but oxygen-rich tissues favor ROS-mediated reactions, including singlet oxygen formation and lipid peroxidation. In plant and algal tissues, where photosynthesis naturally produces oxygen and reactive oxygen intermediates, the probability of oxidative loss may be high unless antioxidant systems are sufficient to protect vitamin D and its precursors [123,124].

Nevertheless, the relationship between oxygen concentration and net vitamin D accumulation has not been characterised systematically across most relevant plant and algal matrices. Its importance should therefore be regarded as mechanistically plausible but incompletely quantified.

Water content and hydration status may also influence the outcome by affecting tissue optics, diffusion of oxygen, membrane organization, and thermal behavior. In mushrooms and other fungal matrices, surface moisture, tissue density, and orientation toward UV-B radiation can alter how much ergosterol is photoconverted. In algae, water-column properties and cellular hydration are inseparable from UV-B exposure because radiation must pass through the aquatic medium before interacting with sterol-containing membranes [129,132].

In technological processing, hydration may also alter surface reflectance, penetration depth, heat transfer, and the stability of vitamin D during subsequent storage. Consequently, water content should be reported together with irradiation conditions and the fresh- or dry-weight basis used to express product concentration.

Taken together, vitamin D accumulation in fungal, algal, and higher-plant systems is controlled by several interacting layers: external UV-B availability, penetration through the tissue surface, sterol precursor abundance, membrane microenvironment, temperature-dependent isomerization, oxygen-mediated degradation, and antioxidant protection. These factors explain why two organisms exposed to similar sunlight may accumulate different vitamin D forms and concentrations. They also explain why controlled production or enrichment requires careful adjustment of irradiation conditions, tissue preparation, and post-exposure handling [123,124,125,126,127,129,130].

These biological differences also complicate comparisons among studies. Reported concentrations should be interpreted in relation to species, tissue, developmental stage, treatment conditions, sample preparation, and the reporting basis used, including fresh-weight, dry-weight, lipid-normalised, or serving-size basis.

Practically, vitamin D formation and UV-B enrichment should be designed around the synthesis–degradation balance. The goal is not simply to expose plant, fungal, or algal material to the highest possible UV-B dose, but to identify conditions under which sterol conversion is maximized while photoproduct formation and oxidative degradation are minimized. This requires attention to wavelength, irradiance, exposure time, temperature, oxygen, tissue thickness, surface area, water content, and antioxidant capacity [131,132,133].

For edible mushrooms irradiated after harvest, this strategy should be described as post-harvest UV-B enrichment. For microalgae and higher plants, the term biofortification should be used according to whether vitamin D content is increased during cultivation through biological or agronomic intervention, rather than solely through post-harvest treatment.

## 7. Analytical Factors and Detection Challenges

After considering the biological and environmental determinants of vitamin D accumulation, it is necessary to address how vitamin D compounds can be reliably detected and quantified in complex higher-plant, algal, and fungal matrices [134].

Accurate quantification of vitamin D in these matrices presents substantial analytical challenges due to extremely low concentrations, often in the ng/g to μg/g range, complex lipophilic matrix interferences from co-extracted phytosterols, chlorophylls, tocopherols, and carotenoids, and structural similarity between vitamin D forms and their photoproducts. Historical methods based on UV spectrophotometry and HPLC with UV detection suffered from limited sensitivity and selectivity, particularly at trace concentrations and in samples containing abundant co-extracted chromophores. Modern mass spectrometric approaches have substantially improved the ability to distinguish vitamin D_2_, D_3_, D_4_, D_5_, and related metabolites in complex biological matrices [126,134].

These difficulties are amplified in plant, algal, and fungal matrices because vitamin D compounds may occur only in trace amounts and may coexist with a broad range of structurally related sterols and lipid-soluble compounds. Because vitamin D is fat-soluble, its extraction, sample stability, recovery, chromatographic behaviour, and detection are closely connected with lipid chemistry [135].

### 7.1. Comparison of Analytical Approaches

The analytical methods used in the literature differ substantially in sensitivity, selectivity, and confidence of compound identification. Historical bioassays provided evidence of vitamin D-like biological activity but could not establish the exact molecular identity of the active compound. Immunoassays may be useful for screening or routine clinical analysis, but antibody cross-reactivity, matrix dependence, and limited validation for plant, algal, or fungal samples restrict their use for identifying uncommon vitamin D analogues. HPLC with UV detection offers chromatographic separation and relatively accessible instrumentation, but co-eluting sterols and other UV-absorbing compounds can compromise selectivity at low concentrations. LC-MS/MS generally provides greater sensitivity and molecular selectivity, although it also requires rigorous chromatographic resolution, matrix-specific validation, and appropriate standards [126,134,135,136,137].

Modern analytical approaches employing liquid chromatography coupled to tandem mass spectrometry (LC-MS/MS), commonly using selected or multiple reaction monitoring, have substantially improved sensitivity and selectivity. Under suitably validated conditions, limits of detection may reach the low pg/g range in selected matrices. However, LC-MS/MS should not be described as providing intrinsically unambiguous identification of vitamin D_2_, D_3_, D_4_, D_5_, or their hydroxylated metabolites. Closely related analogues, positional isomers, epimers, previtamins, and photoproducts may produce similar retention characteristics or fragmentation transitions and may require additional chromatographic or orthogonal confirmation [136,137].

Sample preparation typically requires saponification, liquid–liquid extraction, and solid-phase extraction (SPE) enrichment before LC-MS/MS analysis. Isotope-labelled internal standards, such as [^2^H_6_]-vitamin D_3_, are strongly preferred because they can compensate for extraction losses, variation in ionisation, and analytical drift. However, the internal standard should ideally correspond as closely as possible to the analyte under investigation, and a single D_3_-labelled standard may not fully correct the behaviour of D_2_, D_4_, D_5_, or structurally distinct metabolites [136,137].

### 7.2. Structural Similarity and Compound Identification

The need for highly specific analytical methods follows directly from the structural chemistry of vitamin D. Cholecalciferol and ergocalciferol differ mainly in their side-chain structure: vitamin D_2_ contains a C22–C23 double bond and an additional methyl group at C24, whereas vitamin D_3_ has a saturated side chain. Vitamin D analogues such as D_4_ and D_5_ may be even more difficult to distinguish from related sterol-derived photoproducts. Such subtle structural differences require analytical systems capable of resolving closely related secosteroids rather than merely detecting broad UV-absorbing lipid fractions [138,139].

In plant and algal matrices, analytical uncertainty is amplified by the same photochemical processes that generate vitamin D. UV-B exposure produces not only vitamin D itself, but also previtamin D intermediates, lumisterol, tachysterol, toxisterols, oxidized degradation products, and other sterol-derived compounds. Because these molecules may share similar polarity, absorption properties, or fragmentation behavior, analytical workflows must be designed to distinguish true vitamin D formation from co-eluting or structurally related photoproducts [129,140].

This requirement is particularly important for putative D_4_ and D_5_ identification. A transition compatible with the expected precursor and product ions is not sufficient by itself when authentic standards, retention-time agreement, and qualifying transitions are unavailable. Where standards for uncommon analogues cannot be obtained, compound identity should be reported as tentatively identified.

This distinction is important when comparing naturally exposed samples with materials subjected to controlled UV-B treatment. In natural environments, the UV-B dose is variable and influenced by latitude, season, time of day, ozone concentration, cloud cover, aerosols, altitude, and surface reflection. In controlled irradiation experiments, exposure conditions may be better standardized, but excessive UV-B can still shift the system toward inactive photoproducts rather than vitamin D accumulation. Analytical methods should therefore capture not only the target vitamin D compound, but also the broader photoproduct profile whenever possible [129,140].

### 7.3. Sampling and Reporting Standardisation

A further challenge is sampling standardization. Vitamin D concentrations in mushrooms, algae, and higher plants can vary according to species, tissue type, developmental stage, surface exposure, moisture content, post-harvest handling, UV-B dose, irradiation geometry, temperature, and storage conditions. Without standardized sampling and reporting protocols, comparison across studies remains difficult. For example, fresh weight and dry weight values may differ substantially, and vitamin D concentrations may appear inflated or underestimated depending on whether results are normalized to tissue mass, lipid fraction, serving size, or extracted dry matter [141,142].

Reports should therefore state explicitly whether concentrations are expressed on a fresh-weight, dry-weight, lipid-normalised, culture-volume, biomass, or serving-size basis. When possible, sufficient information should be provided to permit conversion between reporting bases. Values expressed in international units should also be accompanied by mass-based units and the conversion factor applied.

Available radiation data show that UV-related biological outcomes depend strongly on environmental variables such as time of day, season, altitude, cloudiness, atmospheric composition, and surface type. Accordingly, samples collected under natural conditions or exposed experimentally to UV-B cannot be compared reliably unless the relevant exposure parameters are reported. Analytical reports should include information on species, tissue, growth or culture conditions, UV-B wavelength or lamp spectrum, fluence rate, cumulative dose, exposure duration, distance and orientation relative to the source, temperature, moisture, and post-exposure storage [129,134].

For naturally exposed material, date, geographical location, season, approximate exposure conditions, and sampling time should be reported where relevant. For controlled treatment, cumulative UV-B dose is generally more informative than exposure duration alone.

### 7.4. Matrix Effects and Sample Preparation

Matrix effects are especially problematic in LC-MS/MS analysis because co-extracted compounds may suppress or enhance ionization, leading to inaccurate quantification. Chlorophylls, carotenoids, tocopherols, sterols, waxes, and lipid oxidation products may interfere with extraction efficiency or mass spectrometric response. This is why rigorous sample cleanup, matrix-matched calibration, isotope-labeled internal standards, and validated recovery experiments are essential for reliable quantification [136,137].

Plant matrices should not be treated as analytically equivalent to animal serum, fortified foods, or purified supplements. Higher plants contain complex phytochemical backgrounds; algae contain pigments, lipids, sterols, and marine matrix components; and fungi contain ergosterol-rich lipid fractions that may generate multiple UV-induced photoproducts. Each matrix may therefore require adjusted extraction, cleanup, chromatographic separation, and validation procedures [136,137].

Method transfer between matrices should not be assumed to be valid without additional evaluation. A method validated for serum, supplements, or dairy products may perform differently in chlorophyll-rich plant material, saline algal biomass, or ergosterol-rich fungal tissues.

Saponification may improve release of vitamin D from lipid-rich matrices and reduce interference from triacylglycerols, but treatment conditions must be controlled because prolonged heating, oxygen exposure, or inappropriate reagents may contribute to analyte degradation or isomerisation. Liquid–liquid extraction and SPE can increase selectivity, although recovery may vary among free vitamin D, hydroxylated metabolites, glycosides, and uncommon analogues.

### 7.5. Validation Criteria for LC-MS/MS

Reliable LC-MS/MS identification and quantification should include, at minimum, the following validation elements: an authentic analyte standard; an appropriate isotope-labelled internal standard where available; retention-time agreement between the analyte and standard; a validated quantitative ion transition; one or more qualifying ion transitions with acceptable ion ratios; calibration over the relevant concentration range; assessment of selectivity and carry-over; recovery from the actual matrix; within-run and between-run precision; accuracy; limits of detection and quantification; evaluation of ion suppression or enhancement; analyte stability during extraction, storage, and analysis; and analysis of procedural and matrix blanks [136,137,140,141,142,143,144].

For uncommon compounds such as D_4_, D_5_, putative plant analogues, or poorly characterised photoproducts, additional confirmation may be necessary. Potential orthogonal approaches include high-resolution mass spectrometry, alternative chromatographic separation, derivatisation with independent transitions, ultraviolet spectral comparison, nuclear magnetic resonance when sufficient material is available, or confirmation by a second analytical laboratory.

Results should be classified according to identification confidence. A compound detected with an authentic standard, matching retention time, multiple diagnostic transitions, validated recovery, and matrix controls may be described as confirmed identification. A signal based only on nominal mass, a single transition, or comparison with literature fragmentation should be described as tentative identification.

### 7.6. Hydroxylated, Glycosylated, and Other Vitamin D-Related Forms

The detection of hydroxylated vitamin D metabolites in plant matrices presents an additional layer of complexity. In animals, hydroxylated metabolites such as 25(OH)D and 1,25(OH)_2_D are produced through regulated enzymatic metabolism. In plants, however, the presence, origin, and physiological significance of hydroxylated vitamin D-like compounds remain less clear. Analytical confirmation must therefore avoid assuming animal-like metabolic pathways in plant tissues unless enzymatic or mechanistic evidence is available [138,139].

The occurrence of compounds assigned as 25(OH)D or 1,25(OH)_2_D in higher-plant material should not be interpreted as evidence that plants possess vertebrate-like activation pathways. When hydroxylated vitamin D-like compounds are detected in plant tissues, they should be interpreted cautiously and confirmed by high-specificity analytical methods before assigning their metabolic origin or biological function [138,139].

Analytical reports should also distinguish clearly among free vitamin D, esterified forms, glycosides, hydroxylated metabolites, conjugated derivatives, and putative structural analogues. Hydrolysis or deconjugation procedures may convert conjugated compounds into measurable free forms, but the result should not then be reported as the naturally occurring free-vitamin concentration without qualification.

For reports of vitamin D_2_ or ergosterol in higher-plant samples, analytical controls should additionally address possible fungal, endophytic, or epiphytic contributions. Surface sterilisation, microscopy, fungal-marker analysis, cultivation controls, or parallel determination of fungal sterols may help assess this potential source of confounding.

### 7.7. Reference Materials and Interlaboratory Comparability

Another unresolved issue is the lack of harmonized reference materials for plant, fungal, and algal vitamin D analysis. For human serum and some fortified foods, analytical standardization has improved substantially, but higher-plant, algal, and fungal matrices remain less standardized. This limits cross-study comparison and complicates nutritional interpretation, especially when reported concentrations are low, variable, or near detection limits [140,141,142,143,144].

The absence of matrix-specific certified reference materials makes it difficult to distinguish true biological variation from differences in extraction, calibration, chromatographic resolution, and instrumental response. Interlaboratory comparisons and shared quality-control materials would therefore substantially strengthen the field.

Therefore, future studies should move beyond simple detection and toward comprehensive secosteroid profiling. Ideally, analytical workflows should quantify vitamin D_2_, D_3_, D_4_, D_5_, previtamin intermediates, lumisterol, tachysterol, oxidized products, and hydroxylated metabolites in parallel. Such profiling would help determine whether a UV-B treatment increases a nutritionally relevant vitamin D form, generates primarily inactive photoproducts, or promotes oxidative loss [145,146,147].

Comprehensive profiling is particularly important when comparing different UV wavelengths, cumulative doses, treatment geometries, and post-treatment storage conditions. However, the feasibility of simultaneous analysis depends on the availability of authentic standards and validated transitions for each analyte.

Overall, analytical detection is a central determinant of how vitamin D research in fungi, algae, and higher plants is interpreted. Because vitamin D compounds occur at low concentrations, are structurally similar to other sterol-derived molecules, and are embedded in complex biological matrices, reliable conclusions require matrix-specific validation, appropriate compound-identification criteria, and transparent reporting of sample origin, reporting basis, and UV-B exposure conditions. LC-MS/MS currently provides the most appropriate analytical foundation for this field, but its results must be interpreted in relation to chromatographic separation, standard availability, matrix effects, and identification confidence [141,142,143,144].

## 8. Nutritional and Biofortification Implications

The clinical differentiation between vitamin D_2_ and vitamin D_3_ is of nutritional relevance when non-animal dietary sources are considered, because fungal, algal, and higher-plant systems differ in the vitamin D forms they provide and in the strength of evidence supporting their use [138,148].

The nutritional relevance of these sources is reinforced by the global prevalence of vitamin D insufficiency, which remains a major public health problem worldwide. Deficiency may affect people across all age groups, and the number of individuals with insufficient vitamin D status has been estimated at approximately one billion. Limited UV-B exposure, geographical location, seasonality, impaired intestinal absorption, low dietary intake, and lifestyle factors all increase the need for reliable dietary sources of vitamin D [149,150,151].

Comparative supplementation studies generally indicate that vitamin D_3_ is more effective than vitamin D_2_ in raising or maintaining serum 25(OH)D under several dosing conditions. Reported differences have been attributed to variation in binding, metabolism, and clearance, although the magnitude of the difference depends on dose, dosing schedule, baseline status, and study design [138,148].

Biochemical and nutritional sources distinguish cholecalciferol, vitamin D_3_, from ergocalciferol, vitamin D_2_, noting that these two forms differ structurally in the side chain and originate from different substrates. Cholecalciferol is associated mainly with UV-induced conversion of 7-dehydrocholesterol, whereas ergocalciferol is produced through UV irradiation of ergosterol, particularly in fungi and yeasts.

Current non-animal vitamin D strategies range from established post-harvest UV-B enrichment of edible fungi to developing microalgal production and experimental higher-plant biofortification. Their evidence status, analytical requirements, advantages, and limitations are summarized in Table 3.

From a nutritional perspective, this distinction matters because many non-animal vitamin D sources provide mainly vitamin D_2_ rather than D_3_. Mushrooms and yeasts can generate ergocalciferol after UV exposure, whereas algal systems may be more strategically important because some species can produce vitamin D_3_ as well as vitamin D_2_. Therefore, plant-based or vegan vitamin D strategies should consider not only total vitamin D content, but also the chemical form and expected bioavailability of that form [106,152,153,154].

Given the global prevalence of vitamin D insufficiency, there is substantial interest in sustainable non-animal dietary sources. These approaches can be divided into well-established post-harvest UV-B enrichment of fungi, developing microalgal production systems, and experimental higher-plant biofortification or metabolic-engineering strategies [149,150,151].

The need for such strategies is reinforced by geographical and seasonal limitations of UV-B exposure, since vitamin D synthesis depends on latitude, season, time of day, solar zenith angle, altitude, cloud cover, air pollution, and ozone concentration [10,155].

However, these environmental determinants are highly variable and are therefore less suitable for standardised food production than controlled irradiation systems.

### 8.1. Established Strategy: Post-Harvest UV-B Enrichment of Edible Fungi

Edible mushrooms represent the most successful and commercially validated. The high ergosterol content of common culinary mushrooms, including *Agaricus bisporus* at approximately 4–6 mg/g dry weight and *Pleurotus ostreatus* at approximately 3–5 mg/g dry weight, provides an abundant photochemical substrate for UV-B-mediated vitamin D_2_ synthesis. Controlled post-harvest UV-B irradiation can increase mushroom vitamin D_2_ content from low basal concentrations to several hundred or, under selected experimental conditions, several thousand international units per 100 g. Reported yields vary widely with species, sample preparation, exposed surface area, lamp spectrum, irradiance, cumulative dose, moisture, temperature, and the fresh- or dry-weight basis used for reporting. This approach is scalable, cost-effective, and does not require genetic modification, but the irradiation protocol cannot be defined by exposure time alone. Reproducible processing requires reporting lamp spectrum, irradiance, cumulative dose, distance, sample orientation, temperature, humidity, and whether concentrations are expressed per fresh weight, dry weight, or serving size [156,157,158,159,160].

Because irradiation is commonly applied after harvest, this process should be described as post-harvest UV-B enrichment rather than automatically classified as biofortification.

Practical optimisation must also consider tissue orientation and exposed surface area. UV-B acts mainly on accessible tissues, and non-uniform exposure may produce large within-batch differences. Slicing, rotating, or otherwise increasing the exposed area may improve conversion, but excessive treatment can promote discoloration, texture changes, photoproduct formation, or oxidative damage.

Post-treatment stability is another important determinant of nutritional value. Vitamin D_2_ retention may be affected by storage time, temperature, packaging atmosphere, light exposure, drying, freezing, and cooking. Consequently, the vitamin D_2_ concentration measured immediately after irradiation should not be assumed to equal the amount delivered at the point of consumption.

### 8.2. Emerging Strategy: Microalgal Vitamin D Production

Microalgae represent developing candidates for controlled vitamin D production. Reports concerning species such as *Emiliania huxleyi* and selected *Nannochloropsis* and *Chlorella* strains indicate the presence of relevant sterol precursors or UV-B-dependent formation of vitamin D_2_, vitamin D_3_, or both. However, these findings are strongly species- and strain-dependent and require compound-specific analytical confirmation before production capacity is generalised to broader microalgal or cyanobacterial groups [101,102,161,162].

Because taxonomic identity, sterol composition, and vitamin D-forming capacity vary substantially among strains, the suitability of each production organism must be demonstrated experimentally rather than inferred from the general category “microalgae.”

Advantages of microalgal systems include high biomass productivity per unit area, controllable growth conditions, adjustable UV-B exposure, and potential co-production of nutritionally valuable lipids, carotenoids, proteins, and other metabolites. Microalgal cultivation may also be integrated with selected circular-economy processes, although the use of waste streams requires strict control of contaminants and food-safety risks [161,162].

The nutritional relevance of microalgae is further supported by evidence that aquatic food webs may transfer vitamin D-related compounds from planktonic organisms to fish. However, this ecological association does not by itself establish that a given cultivated microalgal strain will produce nutritionally relevant amounts of vitamin D.

Further development requires optimisation of growth phase, biomass density, precursor abundance, light history, UV-B spectrum, cumulative dose, oxygenation, mixing, temperature, and post-irradiation handling. Scale-up must also address self-shading in dense cultures, non-uniform UV penetration, energy demand, contamination control, downstream processing, product stability, and consistency between production batches.

### 8.3. Experimental Strategy: Biofortification of Higher Plants

Biofortification of conventional crop plants faces greater challenges than fungal or microalgal approaches because higher-plant tissues generally contain low or uncertain amounts of appropriate photoreactive 5,7-diene sterol precursors. Additional limitations include restricted UV-B penetration, complex tissue architecture, competing oxidative processes, and the absence of demonstrated vitamin D-specific transport or accumulation mechanisms. Experimental strategies include manipulation of endogenous sterol-biosynthesis and sterol-desaturation pathways to increase appropriate 5,7-diene precursor pools, controlled UV-B supplementation during greenhouse or vertical-farming cultivation, and exploratory post-harvest UV-B treatment of selected higher-plant tissues. These approaches remain experimental and require confirmation that they generate defined vitamin D compounds at nutritionally meaningful concentrations [161,163].

Interventions affecting sterol-biosynthesis or sterol-desaturation pathways require particular caution because altered sterol composition may influence membrane function, growth, development, and plant-hormone biosynthesis. The development of higher-plant biofortification must also account for possible fungal or endophytic contributions to reported vitamin D_2_ or ergosterol. Apparent increases after cultivation or irradiation should not be assigned to plant metabolism unless microbial contamination and associated sterol sources have been adequately excluded [161,163].

### 8.4. Controlled Irradiation, Stability, and Scalability

Environmental variability is directly relevant to these approaches. Natural sunlight varies with latitude, season, time of day, atmospheric ozone, cloud cover, aerosols, and altitude, which makes field-based UV-B exposure difficult to standardize. Therefore, controlled-environment agriculture, greenhouse UV-B supplementation, and post-harvest irradiation may provide more reproducible conditions for vitamin D biofortification than reliance on natural sunlight alone [155,164].

However, controlled irradiation requires optimisation rather than simple maximisation of exposure. Relevant variables include wavelength distribution, irradiance, cumulative dose, treatment time, distance from the source, sample movement, orientation, tissue thickness, surface area, water content, temperature, oxygen availability, and time between irradiation and analysis.

A key practical implication is that biofortification should not be evaluated only by total vitamin D concentration. The chemical form, D_2_ versus D_3_, matrix stability, photoproduct profile, oxidative degradation, serving size, bioavailability, and dietary pattern all determine nutritional value. In populations with limited UV-B exposure or plant-based diets, mushroom-derived D_2_ may be useful, but algal D_3_ could offer a more pharmacokinetically favorable non-animal strategy if production can be scaled and standardized [165,166].

Reported increases should also be evaluated against realistic serving sizes and established dietary intake recommendations. A high concentration expressed per dry mass may have limited practical significance if the consumed portion is small or the product requires extensive rehydration or processing.

### 8.5. Regulatory, Safety, and Food-Production Considerations

Safety and dose control should also be considered. Although biofortified foods are generally designed to provide nutritional rather than pharmacological doses, standardized labeling, controlled production protocols, and monitoring of vitamin D content are necessary to avoid excessive or inconsistent intake [164,167,168].

Regulatory assessment may differ according to whether the intervention involves conventional post-harvest UV treatment, a novel microalgal ingredient, controlled-environment crop production, or genetic modification. Relevant issues may include novel-food status, permitted nutrient claims, maximum fortification levels, specification of the vitamin D form, labelling of UV treatment, stability during shelf life, and evidence supporting the declared concentration [164,167,168].

For microalgae, safety evaluation must also consider strain identity, toxins, heavy metals, microbial contamination, residual culture components, and consistency of biomass composition. For genetically modified higher plants, evaluation must include unintended changes in sterol metabolism, agronomic performance, compositional equivalence, and consumer acceptance [164,167,168].

Functional-food applications will require not only high production yield but also reproducible nutritional delivery after storage, transport, processing, and preparation by the consumer [164,167,168].

The central nutritional conclusion is that UV-B-driven sterol photochemistry can be translated into food production only when precursor availability, spectral dose, matrix characteristics, vitamin D form, analytical confirmation, stability, serving size, and safety are controlled together. Post-harvest UV-B enrichment of fungi is well established, microalgal production is developing, and higher-plant biofortification remains experimental [129,164].

## 9. Future Perspectives and Research Gaps

Future research should distinguish more clearly between the chemical feasibility of vitamin D formation and analytically confirmed production in intact organisms. UV-B-induced B-ring opening is a general property of suitable 5,7-diene sterols, but the efficiency and biological relevance of this process differ substantially among fungal, algal, and higher-plant matrices. Studies should therefore verify the identity, abundance, localisation, and accessibility of the direct precursor rather than infer vitamin D-forming capacity from total sterol content alone [109,111].

Environmental studies should determine how geographical and atmospheric factors influence the UV-B dose actually received by relevant tissues or cultivated biomass. Latitude, season, solar zenith angle, altitude, ozone, cloud cover, aerosols, pollution, and surface reflectance should be integrated with measurements of tissue-level photon penetration rather than considered only as external exposure variables [89,91].

Evidence should continue to be assessed using the standardized categories applied throughout this review: well established for UV-B-induced vitamin D_2_ formation in fungi, developing for vitamin D_2_ and vitamin D_3_ formation in microalgae and other algae, and limited for vitamin D-related compounds in higher plants [113,156,157,158,159,160,161,162].

The quantitative contribution of microalgae and plankton to vitamin D transfer through aquatic food webs remains insufficiently characterised. Future ecological and nutritional studies should combine sterol and secosteroid profiling of primary producers, intermediate consumers, and fish to determine whether specific microalgal taxa contribute materially to vitamin D accumulation in edible aquatic species [161,162].

The relative contributions of precursor photoconversion, thermal isomerisation, photoproduct formation, and reactive oxygen species (ROS)-mediated degradation should be quantified experimentally in defined matrices. At present, these processes are frequently discussed mechanistically but are not measured simultaneously. Controlled studies should therefore determine vitamin D, previtamin D, lumisterol, tachysterol, oxidised products, precursor depletion, antioxidant status, and ROS markers within the same experimental system [123,124].

Optimisation studies should use cumulative spectral dose rather than treatment duration alone and should report wavelength distribution, irradiance, sample distance, orientation, exposed surface area, temperature, hydration, oxygen availability, and post-treatment handling. Response-surface or factorial experimental designs may help identify conditions that maximise a defined vitamin D product while limiting photoproduct formation, oxidative loss, and tissue damage [118,122,129].

Further development should address matrix-specific priorities. Post-harvest UV-B enrichment of edible fungi should be optimised for batch uniformity, storage stability, cooking retention, serving-size relevance, and regulatory compliance. Microalgal systems require strain-specific evaluation of precursor profiles, UV-B penetration, processing, safety, and scalability. Higher-plant biofortification should remain classified as experimental until nutritionally meaningful concentrations and plant-specific origins are confirmed [156,157,158,159,160,161,162,163].

Future nutritional studies should determine whether non-animal vitamin D sources can improve vitamin D status under realistic dietary conditions. Investigations should report the administered vitamin D form, dose, food matrix, serving size, baseline 25(OH)D status, dietary pattern, adherence, and duration of intervention [149,150,151].

Comparative studies should avoid assuming that all D_3_-based strategies are necessarily superior in practical food systems. The nutritional outcome will depend on dose, matrix, stability, intake frequency, bioaccessibility, and product consistency. Mushroom-derived D_2_ remains an established non-animal source, whereas algal D_3_ requires further production, stability, regulatory, and clinical validation before its comparative advantage can be established in routine nutrition [162,165,166].

Safety evaluation should include batch testing, validated concentration measurements, stability over shelf life, realistic intake modelling, and clear labelling of the vitamin D form and amount per serving. The risk of excessive intake from enriched foods is generally lower than that associated with inappropriate high-dose supplementation, but inconsistent production or inaccurate labelling should still be avoided [167,168].

Future analytical work should prioritise matrix-specific secosteroid profiling and apply the identification-confidence categories defined in Section 7. Profiles should include vitamin D_2_, D_3_, D_4_, D_5_, relevant previtamins, photoproducts, oxidised products, hydroxylated metabolites, and conjugated forms where suitable standards are available [137,141,142,143,144,145,146,147].

Minimum reporting standards should include organism and strain identity, tissue or biomass type, developmental or growth stage, sampling basis, fresh-weight, dry-weight, lipid-normalised, culture-volume, biomass, or serving-size basis, as applicable, UV spectrum, irradiance, cumulative dose, exposure geometry, temperature, moisture, oxygen conditions, interval before extraction, and storage conditions. Adoption of common reporting standards would substantially improve interstudy comparison [126,128,129].

Potential physiological functions of vitamin D-related compounds in higher plants remain speculative. Future work should first establish endogenous compound identity and localisation, exclude microbial sources, and determine whether concentrations change reproducibly with genotype, development, environmental treatment, or disruption of defined sterol pathways. Only then should candidate binding proteins, transport systems, or signalling functions be investigated [117,130].

Hydroxylated or glycosylated vitamin D-related compounds detected in plants should not be assigned vertebrate-like metabolic origins or functions without direct enzymatic and molecular evidence. The distinction among free vitamin D, conjugates, 25(OH)D, 1,25(OH)_2_D, other hydroxylated metabolites, and putative analogues should be maintained consistently [138,153].

Evolutionary interpretations should remain explicitly hypothetical. Comparative sterol biochemistry, palaeoenvironmental modelling, and phylogenetically broad analyses may clarify when photoreactive sterols arose and whether vitamin D-related products provided any selective advantage, but the non-enzymatic occurrence of a photoproduct does not itself demonstrate an evolved physiological function [111,117].

Environmental monitoring should be coupled with biological measurements before conclusions are drawn about changing vitamin D formation potential. Changes in incident UV-B may not translate directly into product accumulation because tissue optics, precursor availability, acclimation, antioxidants, and degradation processes can also change [89,91].

Microalgal photobioreactor development should focus on strain selection, precursor profiling, culture density, mixing, self-shading, oxygenation, UV-B delivery, energy use, harvesting, extraction, product retention, contaminant control, and regulatory status. Co-production of lipids, carotenoids, proteins, or other metabolites may improve economic feasibility, but interactions between UV-B treatment and these valuable components should also be assessed [161,162].

Controlled production systems are likely to provide greater reproducibility than reliance on natural sunlight. Nevertheless, post-harvest UV-B enrichment of fungi should be distinguished from agronomic biofortification during crop growth and from experimental post-harvest treatment of higher-plant foods [156,157,158,159,160,164].

Human intervention studies should prioritise products with fully confirmed compound identity, reproducible dose, demonstrated storage stability, and realistic serving sizes. Trials should compare enriched foods with reference D_2_ or D_3_ preparations while accounting for matrix effects, intake frequency, baseline status, and changes in serum 25(OH)D [165,166].

The interpretation of clinical outcomes should account for dietary intake, supplement use, sun exposure, season, age, body composition, absorption disorders, and other determinants of vitamin D status [149,150,151,152,153].

## 10. Conclusions

Vitamin D formation is not restricted to animal tissues, but evidence for its occurrence and nutritional relevance differs markedly among non-animal systems. UV-B-induced conversion of fungal ergosterol to vitamin D_2_ is well established and underlies commercially applicable post-harvest enrichment of edible mushrooms. Microalgae represent an developing platform for vitamin D_2_ and vitamin D_3_ production, but strain selection, analytical confirmation, scalability, safety, and stability require further study. Evidence for vitamin D formation and nutritionally meaningful accumulation in higher plants remains limited, methodologically heterogeneous, and potentially confounded by fungal or endophytic contributions [161,162,163].

The central chemical sequence is straightforward: an appropriate 5,7-diene sterol is converted by UV-B to previtamin D, which isomerises thermally to vitamin D and may undergo further photochemical or oxidative degradation. The biological and nutritional interpretation is less straightforward because precursor abundance, tissue accessibility, matrix composition, irradiation conditions, analytical selectivity, and product stability all influence the measured outcome [43,123,129].

Accordingly, chemical plausibility should not be treated as evidence of natural occurrence, and detection should not automatically be interpreted as proof of physiological function or nutritional significance. Reliable conclusions require validated, matrix-specific analytical methods, clear classification of evidence, standardised reporting, and distinction among fungi, algae, and higher plants [117,126,141].

The most immediate practical priority is optimisation and standardisation of UV-B-enriched fungal foods. Future progress will depend less on expanding the number of reported detections and more on improving compound confirmation, interlaboratory comparability, process reproducibility, nutritional evaluation, and transparent acknowledgement of current limitations [129,141,147].

## Figures and Tables

**Table 1:** Principal vitamin D forms, chemically appropriate precursor sterols, reported biological sources, structural characteristics, and current strength of evidence.

Vitamin D Form and Precursor	Reported Source or System	Molecular Weight and Structural Feature	Standardized Evidence Category and Interpretation
Vitamin D_2_ (ergocalciferol) Ergosterol (provitamin D_2_)	Fungi and yeasts; reported in selected algal and higher-plant matrices	396.65 g/mol C22–C23 double bond and C24 methyl group	Well established in UV-B-exposed fungi and yeasts. Evidence in algal matrices is developing, whereas evidence in higher plants is limited and requires organism- and method-specific confirmation.
Vitamin D_3_ (cholecalciferol) 7-Dehydrocholesterol (provitamin D_3_)	Animals; reported in selected microalgae, other algae, and higher-plant matrices	384.64 g/mol Saturated side chain lacking the additional C22–C23 double bond and C24 methyl group present in vitamin D_2_	Well established in animals. Evidence in algae is developing, whereas evidence in higher plants is limited and heterogeneous.
Vitamin D_4_ (22-dihydroergocalciferol) 22,23-Dihydroergosterol (provitamin D_4_)	Fungi and experimental sterol-photolysis systems; occurrence in other organisms is less well characterised	Approximately 398.67 g/mol Saturated C22–C23 bond relative to vitamin D_2_	Experimental. The precursor–product relationship is chemically defined, but biological distribution and nutritional relevance remain insufficiently characterised.
Vitamin D_5_ (sitocalciferol) 7-Dehydrositosterol (provitamin D_5_)	Primarily experimental photochemical systems; reported relevance to phytosterol-containing matrices	Approximately 412.70 g/mol C24 ethyl substituent	Limited. Chemical formation is plausible from the appropriate 5,7-diene precursor, but natural occurrence and nutritional relevance in higher plants remain weakly supported.

**Table 2:** Representative reports of vitamin D-related compounds in fungi, algae, and higher plants, including analytical method, reporting basis, and evidence level.

Organism and Material	Reported Compound and Amount	Reporting Basis and Context	Analytical Method	Evidence Category, Nutritional Relevance, and Main Limitation
Higher plant Solanum glaucophyllum leaves	Vitamin D_3_-related activity and 1,25(OH)_2_D_3_-related compounds, including glycosylated forms Approximately 3–7 μg/g in selected studies	Dry weight; compound identity and reporting basis vary among studies Natural plant material	Bioassay and HPLC-based methods	Limited. Nutritional relevance cannot be established because molecular form, bioavailability, and serving basis are unclear. Main limitation: free, glycosylated, and hydroxylated forms are not consistently distinguished.
Higher plant Cestrum diurnum	Vitamin D_3_-related glycosides or hydroxylated metabolites Qualitative or semi-quantitative	Basis not reported consistently; fresh-weight, dry-weight, or serving basis cannot be assigned Natural plant material	Bioassay and mass-spectrometric approaches	Limited. Nutritional relevance cannot be quantified. Main limitation: exact molecular form and concentration remain uncertain.
Higher plant Trisetum flavescens	Vitamin D_3_-related glycosides Qualitative or semi-quantitative	Basis not reported consistently Natural material associated with calcinogenic activity	HPLC and biological evidence	Limited. Nutritional relevance cannot be quantified. Main limitation: compound identity and glycosylation state are not uniformly confirmed.
Higher plant Allium ursinum	Vitamin D_2_ Trace to low amounts	Reporting basis not consistently available Natural plant material	LC-MS/MS	Developing. Nutritional relevance remains uncertain because concentrations are low and serving basis is unclear. Main limitation: fungal, endophytic, or epiphytic contributions must be excluded.
Fungus Agaricus bisporus and other edible mushrooms	Vitamin D_2_ From low basal amounts to >40,000 IU/100 g under selected irradiation conditions	Fresh weight, dry weight, or serving-size basis, depending on the study Controlled post-harvest UV irradiation	HPLC and LC-MS/MS	Well established. Nutritional relevance may be high per edible serving. Main limitation: comparisons require harmonized units and irradiation conditions.
Microalga Emiliania huxleyi	Vitamin D_2_ and vitamin D_3_ Variable and UV-dependent	Dry biomass, fresh biomass, culture volume, or serving basis must be specified Natural or experimentally irradiated cultures	Chromatographic and mass-spectrometric methods, including LC-MS/MS in selected studies	Developing. Nutritional relevance remains uncertain because values are not consistently reported per edible biomass or serving. Main limitation: compound-specific confirmation is required.

**Table 3:** Comparative status of non-animal vitamin D production, biofortification, and post-harvest UV-B enrichment strategies.

Strategy and Biological System	Vitamin D Form and Intervention	Evidence Category	Key Analytical Requirements	Practical Advantages and Principal Limitations
Post-harvest UV-B enrichment Edible mushrooms and other fungal foods	Mainly vitamin D_2_ Controlled UV-B treatment after harvest	Well established	Validated HPLC or LC-MS/MS; fresh- or dry-weight or serving-size basis; UV-B dose and storage conditions	Advantages: high ergosterol availability, scalability, and no requirement for genetic modification. Limitations: uneven exposure, photoproduct formation, storage and cooking losses, and need for dose standardisation.
Controlled microalgal production Microalgal and phytoplankton cultures	Vitamin D_2_ and/or D_3_, depending on species Cultivation with controlled UV-B exposure	Developing	Authentic standards; validated LC-MS/MS; biomass-normalised results; confirmation of precursor and product identity	Advantages: high biomass productivity, controllable cultivation, and potential co-production of valuable compounds. Limitations: species-dependent yields, limited standardisation, scale-up challenges, safety concerns, and regulatory uncertainty.
Controlled-environment biofortification Higher plants during growth	Reported vitamin D_2_, vitamin D_3_, or related compounds Greenhouse or vertical-farming UV-B supplementation	Experimental	Matrix-specific confirmation; exclusion of fungal or endophytic sources; clear classification of compound form	Advantages: potential integration with existing crop-production systems. Limitations: low or uncertain precursor abundance, tissue optical barriers, oxidative degradation, and inconsistent evidence.
Metabolic engineering Higher plants or microalgae	Targeted vitamin D_3_ or another defined form Modification of sterol-biosynthesis pathways	Experimental	Confirmation of sterol-pathway changes, compound identity, stability, and nutritional relevance	Advantages: potential increase in precursor pools and greater control of product profile. Limitations: agronomic effects, unintended sterol changes, regulatory requirements, safety assessment, and consumer acceptance.
Post-harvest UV-B treatment of plant foods Leaves, fruits, or other plant tissues	Reported or putative vitamin D-related compounds UV-B exposure after harvest	Limited	Orthogonal confirmation of compound identity; distinction from photoproducts and microbial sources	Advantages: technologically simple in principle. Limitations: uncertain nutritional yield, possible tissue damage, oxidation, and product instability.

## Data Availability

No new data were created or analyzed in this study. Data sharing is not applicable to this article.
